# The Development of Game-Based Digital Mental Health Interventions: Bridging the Paradigms of Health Care and Entertainment

**DOI:** 10.2196/42173

**Published:** 2023-09-04

**Authors:** Lauri Lukka, J Matias Palva

**Affiliations:** 1 Department of Neuroscience and Biomedical Engineering Aalto University Espoo Finland; 2 Neuroscience Center, Helsinki Institute of Life Science University of Helsinki Helsinki Finland; 3 Centre for Cognitive Neuroimaging, School of Psychology and Neuroscience University of Glasgow Glasgow United Kingdom

**Keywords:** digital mental health interventions, development frameworks, gamification, game-based interventions, intervention development, mental health, paradigms, serious games

## Abstract

Game elements are increasingly used to improve user engagement in digital mental health interventions, and specific game mechanics may yield therapeutic effects per se and thereby contribute to digital mental health intervention efficacy. However, only a few commercial game–based interventions are available. We suggest that the key challenge in their development reflects the tension between the 2 underlying paradigms, health care and entertainment, which have disparate goals and processes in digital development. We describe 3 approaches currently used to negotiate the 2 paradigms: the gamification of health care software, designing serious games, and purpose shifting existing entertainment games. We advanced an integrative framework to focus attention on 4 key themes in intervention development: target audience, engagement, mechanisms of action, and health-related effectiveness. On each theme, we show how the 2 paradigms contrast and can complement each other. Finally, we consider the 4 interdependent themes through the new product development phases from concept to production. Our viewpoint provides an integrative synthesis that facilitates the research, design, and development of game-based digital mental health interventions.

## Introduction

### Background

Digital mental health interventions (DMHIs) are actively developed in response to the growing mental health crisis [1]. Some of the new interventions [2-6] use elements from entertainment games to improve user engagement and intervention effectiveness [7,8]. Using a game-based approach to pursue health-related aims requires developers to have competencies in both health care and game development; however, the 2 paradigms have considerable disparity in their goals. In health care, health-related aims are pursued by minimizing user interaction with the intervention, whereas entertainment products, largely oblivious to their health effects, seek to maximize user interaction with the content. This difference has led to specialized practices, institutions, and business models that are not readily compatible and challenge interdisciplinary development, which Mathews et al [9] succinctly describe as follows:

The “fail fast, fail often” mantra espoused by technology startups is frustrated by the confusing regulatory landscape of healthcare. This cultural clash is further exacerbated by the cautious, stepwise, and time-consuming process of healthcare innovation that is grounded in the risk-averse clinical principle of “first, do no harm.”

Existing models of intervention development [10-13], although comprehensive, do not sufficiently address the tension between health care and entertainment in the development of game-based DMHIs (gDMHIs). In this paper, we describe the tension and offer a framework to facilitate new product development and implementation.

### The Treatment, Engagement, and Implementation Gaps in Digital Interventions

Mental disorders are common and cause distress and burden to both individuals and society [14]. However, most people with mental disorders remain untreated. The median treatment gap, defined as the difference between the prevalence of a disorder and those treated for it, is >50% for many disorders [15]. For instance, only a fourth of those living with anxiety disorders receive any treatment, with 1 (10%) in 10 receiving possibly adequate treatment [16]. The mental health care system is globally unable to offer sufficient interventions to address patients’ needs.

Solutions to the mental health burden are increasingly sought from scalable digital interventions. The evidence of their effectiveness for both depression and anxiety is already substantial [17-19], and their effectiveness compares to that of face-to-face interventions [20]. Moreover, the treatments display a considerable dose-response relationship [17,18,21,22]: greater user interaction with the software-based intervention, that is, engagement, is associated with higher intervention effectiveness. However, digital interventions suffer from an engagement gap. High dropout rates and low user engagement are observed in digital interventions, particularly in self-guided programs [17,23-25], where 40% of the participants may drop out before completing a fourth of the intervention [23], and only 0.5% to 28.6% complete the entire intervention or continue its use [26]. As engagement is related to intervention effectiveness, there is a substantial need to ensure that users find the interventions interesting, motivating, and meaningful.

The engagement gap is also an indication of the implementation gap: researchers and developers struggle to transfer the effectiveness established in clinical settings to real-world environments [27]. The effectiveness of DMHIs in ideal and controlled research settings (efficacy trials) is almost double that in real-world settings (effectiveness trials) [18,28]. The intervention’s clinical effectiveness in optimal circumstances does not guarantee real-world impact, which calls for paying more attention to how the interventions are designed and developed with both users and clinicians in mind.

### Game-Based Approaches Seek to Increase Intervention Engagement and Effectiveness

Game-based approaches are increasingly developed with the ambition to improve user engagement with and the effectiveness of DMHIs [7,8]. This rationale reflects the immense popularity of digital entertainment games. Playing video games is a hobby for 71% of American youth and 65% of adults [29], and globally, there are >2.7 billion players [30]. Although youth and young adults are the most active players, the average player is aged 38 years, and those in their 30s still play, on average, >6 hours a week [31]. Digital games are a familiar, accessible, attractive, and engaging medium for the general audience.

Game-based interventions are currently developed for numerous psychiatric disorders, including depression [3], anxiety [2], attention-deficit/hyperactivity disorder (ADHD) [5], and autism spectrum disorder [6], and serious mental illnesses, including schizophrenia and bipolar disorder [4]. Fleming et al [8] suggested that game-based interventions can extend treatment reach, thus helping close the treatment gap and improve user engagement and intervention effectiveness. There are numerous game-based interventions in development [32], but only a few are in the market. To support their development, there is a considerable need for frameworks that address the particularities of the game-based medium and facilitate interdisciplinary collaboration.

### The Aim of This Paper

There is a substantial need for DMHIs that are engaging, effective, and feasible, and game-based approaches have potential in this regard. In this paper, we offer an interpretive synthesis that integrates and contrasts the literature in 2 fields: health care and entertainment. First, we argue that the 2 fields are in tension, which challenges the development of game-based interventions. Then, we discuss how to negotiate the tension. We focus on 4 themes, namely the target audience, engagement, mechanisms of action, and effectiveness (TEME) framework. We conclude the discussion by reflecting on the themes through new product development phases. Aimed at researchers, developers, and designers working with game-based interventions, our paper facilitates interdisciplinary collaboration and the wise use of game-based elements to ameliorate mental health.

## The Tension Between Health Care and Entertainment

### The Paradigms Address Different Needs

We suggest that the engagement gap with DMHIs reflects a tension between health care and entertainment, which becomes especially apparent in game-based interventions. Our approach is built on the notion of the differentiation of society into subsystems such as law, economy, politics, religion [33,34], health care, and entertainment. The thorough history of the 2 paradigms is beyond the scope of this paper, but their differences can be summarized in the code that they specialize in. Health care is concerned with the code of health, focusing on identifying and classifying sources of disability and seeking remedies for them [35]. Moreover, health care has legitimized priority over the domain of health, manifesting in the authority of certified clinicians overviewed by regulatory bodies. Meanwhile, entertainment, also described as “audience-centered commercial culture” [36], is a societal subsystem focusing on the code of leisure. The value of entertainment manifests through its ability to attract and captivate the attention of the audience over time in the theater and through literature, music, and games. The freedom of expression prevails in these domains because of the low risk to audiences’ health. The subsystem codes are associated with underlying universal human needs. According to the notions proposed by Max-Neef et al [37], health care addresses the existential needs of subsistence and protection by curing and helping, whereas entertainment responds to the needs of idleness, creation, and identity by offering fantasies, relaxation, and opportunities to have fun both alone and together with others.

The organizations catering to the same need differentiate into industries that share a high-level *raison d’être* and have specialized concepts, regulations, structures, competencies, and methodologies to support their work. This specialization both increases the effectiveness in addressing the underlying human needs *and* creates distance from other societal subsystems. Refocusing on game-based mental health interventions, they can be viewed as a combination of 2 domains: the entertainment game industry, which has developed specialized skills in digital art and animation, programming, project management, storytelling, game design, and media business, and mental health care, which focuses on psychiatric diagnoses and their etiology and treatment. To summarize, health care practices are not intended to craft digital entertainment and vice versa. The differences between DMHIs and entertainment games are illustrated in the Table 1.

**Table 1:** Differences between the paradigms of health care and entertainment are exemplified through digital mental health interventions (DMHIs) and digital entertainment games.

	DMHI	Digital entertainment games
Underlying paradigm	Health care	Entertainment
Paradigm code	Health	Leisure
Underlying need	Alleviating disorders and increasing well-being	Offering enjoyment, relaxation, and social connection
Users	Patients, clients	Consumers, players, fans
Goal	Introducing behavioral change and alleviating symptoms	Captivating and entertaining the player
The scientific base	Extensive research base	Growing research base
Categorized by	Therapeutic modality (eg, CBT^a^)	Game genre (eg, FPS^b^)
Evaluated by	Efficacy and safety	Game experience, business metrics, and reviews
Initiative to use	Often recommended by a health care professional	Chosen by the player from a wide variety of alternatives
Entry to market	High threshold: clinical evidence, regulations, and gatekeepers	Low threshold: global digital marketplaces
Availability	Growing number of interventions	High number of commercial games
Purchase	Insurance often pays for the service.	The customer pays for the service.
Business model	B2B^c^	B2C^d^

^a^CBT: cognitive behavior therapy.

^b^FPS: first-person shooter.

^c^B2B: business to business.

^d^B2C: business to consumer.

We are not aware whether the tension between health care and entertainment would have been previously framed in this way. However, the disparate goals of health care and entertainment have attracted prior attention. Yardley et al [38] discussed the need for digital interventions to promote “effective engagement” rather than merely pursuing an increase in user interaction. They highlighted the need to establish a causal connection between user interaction with the intervention and the intended behavior change. Exploring gamified information systems, Liu et al [39] described the phenomenon as “meaningful engagement”: the intervention needs to reach its experiential outcomes—to be sufficiently enjoyable and feasible—to reach its instrumental outcomes of behavior and symptom changes. Furthermore, Siriaraya et al [40] separated “game value” from “therapeutic value,” highlighting the difference between the 2 paradigms. These considerations emphasize how engagement and effectiveness are distinct, and we suggest that this relates to the 2 paradigms and their codes.

### Differentiating Among Gamified Interventions, Serious Games, and Purpose-Shifted Entertainment Games

**Figure 1:**
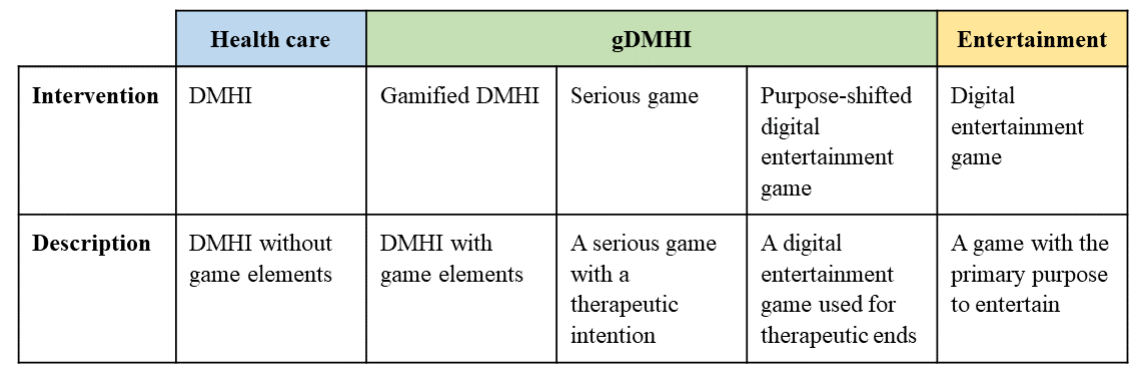
Three categories of game-based digital mental health interventions (gDMHIs) are positioned between health care and entertainment. DMHI: digital mental health intervention.

DMHIs with and DMHIs without game elements are actively developed, and the former particularly negotiates the underlying paradigms of health care and entertainment. We classified game-based interventions into the following 3 categories: gamified DMHIs, serious games, and purpose-shifted digital entertainment games. We refer to them collectively as gDMHIs, “game-based interventions” in short*.* As the intervention adapts more elements from entertainment games, on the one hand, its gameness [41] increases, and, on the other hand, its intentional therapeutic functionality transforms (Figure 1).

At one end of the continuum, DMHIs pursue health benefits and use minimal game-based elements. At the other end, there are digital entertainment games that may have incidental health benefits to their primary aim of capturing the interest and attention of the player. The former can be described as functional software that serves a particular purpose, whereas the latter is built to create experiences that are valuable in their own right [42,43]. The design of digital entertainment games is a specialized domain differentiated from traditional, functional software development [44,45], which is also reflected in the concepts used. Functional software has users, and its evaluation focuses on usability and user experience, whereas games are evaluated based on their playability and game experience [46].

Between health care and entertainment lie 3 categories of gDMHI. When game design elements are incorporated into a DMHI, the intervention becomes gamified [47]. The 2 most common rationales for using gamification in health care are improving user engagement and the effectiveness of the intervention, and the most common game elements include levels, progress feedback, points, rewards, narration, and personalization [7]. The incorporated game elements should not be superficial and exogeneous to the designed intervention, but synergistic with the experience the intervention intends to create [48]. In addition, gamification is not limited to the features visible to the user but can include the use of game design principles and methods in design and development [47].

In contrast to gamified applications, serious games are full-fledged games that are used for purposes beyond entertainment [47,49], often in education, health care, and organizational development. Gamified applications and serious games are frequently differentiated in the research literature [50], but in practice, the line between the two is not easy to draw. To distinguish between gamified and full-fledged game interventions, Liu et al [39] suggested that gamification incorporates game elements on top of real-world systems without sacrificing their functionality, whereas serious games are separate from real-world systems. Thus, a gamified intervention may more closely resemble and augment functional applications while retaining the instrumental functions of the system, whereas a full-fledged game often includes a fictional world for the player to immerse in.

To further clarify, we differentiated game-based interventions from “serious games for health” serious games used within the health care system by clinicians and patients for therapeutic and educational purposes [51,52]. Not all game-based approaches within health care are interventions: only those that prevent, assess, and help manage disorders are interventions, in contrast to software with educational and information management aims [53].

Whereas gamified and serious game DMHIs are intentionally designed for therapeutic purposes, purpose-shifted games take an alternative approach. These are games designed for entertainment purposes but used for serious, therapeutic purposes, either with or without modifications [54]. The rationale for this approach lies in the finding that playing digital entertainment games may have a positive impact on well-being regardless of the design and use intention of the games [55,56]. Positive effects arise, for instance, from the users’ connection with other people, which that alleviates loneliness and creates meaningful relationships; the games’ cognitive demands, which train attention, perception, and executive functions; and the positive, eudaimonic experiences offered by the games [57,58].

The forte of the purpose-shifted approach is that commercially available video games are accessible, affordable, and of high quality compared with many designed gamified interventions and serious games. Consequently, this approach is quite popular. Recent reviews found that 14 of 27 interventions for depression [3] and 13 of 28 interventions for anxiety [2] used a purpose-shifted approach, and the rest of the interventions were designed. However, the approach also comes with a considerable downside: researchers have limited opportunities to continue intervention development because they do not control the intellectual property, code, art assets, or delivery of the game.

Differentiating among gamified games, serious games, and purpose-shifted entertainment games exhibits the variance in gDMHIs. Moreover, it contributes to understanding the developer’s approach and position between the 2 underlying paradigms: is the intervention a functional application to which game elements are added, an entertainment game whose purpose is shifted, or a tailor-made serious game? Because game-based interventions draw from both health care and entertainment, there is a need to consider both perspectives in their development.

## Bridging the Paradigms of Health Care and Entertainment

### The TEME Framework

#### Overview

Interventions are intended for a particular audience who, by interacting with the interventions, hopefully, achieve positive changes that are explained by the interventions’ mechanisms of action [10-13]. To contrast and discuss the paradigms of health care and entertainment, we focus on 4 interrelated themes that are necessary, but not sufficient, for the development of new products (Table 2). We acknowledge that other aspects are also necessary, including privacy [59,60] and integration with existing infrastructure [61]. However, we consciously focus on the 4, as they are illustrative of the differences between and strengths of the 2 paradigms and serve to solve the tension between them.

**Table 2:** The target audience, engagement, mechanisms of action, and effectiveness (TEME) framework for game-based intervention development.

Theme	Key question	Health care	Entertainment
Target audience	Who is the intervention engaging and effective for?	Health indication and user characteristics frame the target audience.	Differences in game preferences create various audiences.
Engagement	Does the user want to interact with the intervention?	Engagement is associated with effective mechanisms of action.	Subjective and objective engagement are measured.
Mechanisms of action	What explains the intervention effects?	The mechanisms of action are evidence based.	The mechanisms of action are synergistic with their game-based implementation.
Effectiveness	What does the intervention achieve?	The intervention helps achieve behavioral change and symptom reduction.	Interaction is satisfying and fulfills the players’ psychological needs.

#### Target Audience: Who Is the Intervention Engaging and Effective for?

Intervention development begins with the target audience’s needs [62]. In mental health, there are 2 main ways in which user needs are conceptualized: a diagnostic approach and transdiagnostic approach [63]. The former frames the need through diagnostic taxonomies, and the latter focuses on a particular symptom or health-related behavior. However, both approaches frame the intervention in terms of a health indication, and it is the health care paradigm that provides the initiative; goal; and, consequently, methods for evaluating the intervention’s success.

Health indications, such as ADHD, schizophrenia, and depression, create the initial frame for the target audience. However, the actual target market is a subpopulation of this total addressable market influenced by several implicit factors. Users’ age, gender, education, personality, and prior experiences with mental health services and the severity of their symptoms influence user engagement [64]. Knowing these background factors provides an opportunity to target audiences that favor digital interventions and to extend the intervention reach to new and underserved audiences through inclusive and participatory design [65]. Although our discussion emphasizes the end user, the intervention’s target audience may also include other stakeholders, such as therapists in case of interventions that blend web-based and face-to-face therapies [66].

Besides the factors influencing DMHI use in general, some aspects influence user engagement with gDMHIs specifically. This reflects the considerable differences in users’ game preferences [67]. Appreciating the divergence in player motivations goes back to Bartle [68], who noticed that the players of multiuser dungeons (an obsolete multiplayer game genre) engage with the games for divergent reasons and in different ways. On the basis of “a long, heated discussion,” he identified the following 4 motivations to play: *achievers* play to pursue game-related goals, *explorers* seek to understand the fictional game world, *socializers* focus on role playing and interacting with others, and *killers* compete for dominance through conflict. Bartle’s [68] insight was that players engage in an apparently similar activity for widely different reasons and use the affordances of the game to serve their preferences. Since his original work, player motivations have been explored further. A review of 12 player typologies found that there is surprising conceptual uniformity in the categorizations, concluding with 5 primary player motivations, adding immersion¸ which is concentration on the story, fantasy, and narrative, to Bartle’s original 4 [69]. In addition to affecting *how* games are played, game preferences influence *which* games are played. Using survey data, Mandryk and Birk [70] classified players into 4 categories: those who appreciate single-player games, those who appreciate first-person shooter or action games, those who appreciate casual games, and those who appreciate most genres. Moreover, the researchers found that their categorization was not associated with depression, indicating that people with depression appreciate various genres. Thus, the game genre decision partly defines who finds the genre attractive and engaging.

We advocate an integrative approach to the target audience. The health indication provides a rationale for the intervention and limits the target audience. In addition to the factors influencing engagement with DMHIs, the chosen game genre influences who are drawn to the game-based intervention, continue to engage with it, and hence gain a positive impact from the interaction. Understanding the target audience allows developers to identify and cater to the variance in the users, their needs, play styles, and preferences and to remove frictions that prevent players from enjoying the intervention as much as they expect to.

#### Engagement: Does the User Want to Interact With the Intervention?

The user’s motivation to interact with the gDMHI is to gain certain benefits and enjoy their time while doing so. According to concepts proposed by Siriaraya et al [40], the “therapeutic value” of the intervention is a function of its “game value”: the more experientially pleasurable the intervention is, the more likely the player is to continue to interact with it. These 2 concepts are related through the behavioral change that the interaction invites. The capability, opportunity, motivation, and behavior (COM-B) system describes 3 antecedents for behavioral change [12]. To build the user’s capabilities, opportunities, and motivation, the intervention can, for instance, educate, persuade, train, and model new behaviors, which we call the mechanisms of action. Behavioral change can lead to symptom change [13], although often after a significant delay of days and weeks, which should motivate attention to the player experience.

Mere interaction with the intervention, however, is not sufficient, which Yardley et al [38] highlighted through the concepts of microlevel and macrolevel engagement. For instance, an exergame delivers its effects when the user trains with it, exhibiting microlevel engagement. This may turn into macrolevel engagement with the broader goals of the intervention when the intervention teaches the user healthy exercise routines and encourages the transfer of new behavior outside the immediate interaction. Thus, unlike commercial entertainment games, whose success is often closely tied to microlevel engagement, gDMHIs are driven by 2-tier engagement, and a successful intervention becomes obsolete once the player has internalized the intended change, which is a considerable digression from the entertainment paradigm. Subjectively, microlevel engagement may be experienced as a reduced effort to interact with the intervention. Subjectively, microlevel engagement may be experienced as a reduced effort to interact with the intervention [71]. This is also what the player may expect from a game-based approach: when the intervention is introduced through the notions of “game” and “play,” they allude to interactions that are more pleasurable than those with solutions more strongly associated with a functional approach.

To design for microlevel engagement, the concept needs a clear definition and metrics. Here, we turn to Perski et al [72], who provide a useful definition: “(1) the extent (eg, amount, frequency, duration, depth) of use and (2) a subjective experience characterized by attention, interest and affect*.*” The 2 sides represent complementary aspects of engagement, objective and subjective aspects, which require different approaches in their evaluation [73,74]. The subjective criteria may be pursued through qualitative research, such as user interviews, think-aloud protocols, and focus groups that provide rich and detailed qualitative data on engagement [75], or swiftly through questionnaires [76]. Subjectively, high levels of engagement with gDMHIs may be experienced as immersion, where the player becomes unaware of the mediating technology, perceiving the interaction as unmediated [77]. The phenomenon is also known as presence, a sense of being “in the game.” It has many similarities to the notion of flow, where the subject is absorbed in the task and becomes unaware of their surroundings [74,78,79]. This pleasurable mental state is the experiential value players seek from the game and manifests as a sense of “want to” rather than “have to” engage with the game [80].

To complement the evaluation of a subjective sense of engagement, game analytics can provide objective indicators of engagement. Among the strongest and most valuable tools in commercial entertainment game development, game analytics provides proof, also in business terms, that the target audience enjoys spending time with the game [81]. User analytics offers insights into microlevel engagement by measuring the time spent with the intervention, accompanied by metrics of retention, that is, players returning to the intervention.

Entertainment games are a medium focused on building engagement that is experienced as immersion. This provides the developers with a user-centered goal that can be evaluated both subjectively and objectively. The health care paradigm complements the approach by ensuring that evidence-based mechanisms of action are in place so that user interaction contributes to the expected health benefits.

#### Mechanisms of Action: What Explains the Intervention Effects?

##### Overview

Mechanisms of action explain how and why the intervention achieves the intended change in the user’s behavior and symptoms [10]. They have also been called “mechanisms of change” [13], and “intervention functions” [12], and they include sharing knowledge, building motivation, changing beliefs, modeling new behaviors, and persuading change. In game-based interventions, these rationales are given a digital form through their game-based implementation. Next, we describe and contrast 3 common and distinct approaches to game-based interventions: adapting existing psychotherapies, offering cognitive training, and encouraging physical exercise (Table 3).

**Table 3:** Three approaches to game-based interventions exhibit the potential synergy between health care rationale and its game-based implementation.

Approach	Mechanisms of action	
	Health care: “what”	Entertainment: “how”
Adapting existing psychotherapies	Behavior change through an increase in mentalization capabilities	Narrative and story-driven implementations
Offering cognitive training	Training cognitive functions such as attention	High-paced action games
Encouraging physical exercise	Increased physical activity	Virtual reality, augmented reality, and fitness games

##### Adapting Existing Psychotherapies

Numerous existing psychotherapies have been adapted as gDMHIs, the most prominent of which is cognitive behavioral therapy [2,3]. There are numerous benefits to using an existing therapy to guide the development work: it provides a theoretical foundation, a rationale on how and why the intervention should work, credibility with stakeholders, and legitimacy for the work. Despite the differences between psychotherapies, their effects seem curiously similar [82]. It has been suggested that there are common factors that explain their effectiveness [83], although the research is ongoing [84]. Leiman [85] conceptualized that all psychotherapies seek to change the patient’s position toward their challenges. When the client begins the therapy process, they are in an object position to their problem. Through therapy, they gain an understanding of their problem and capabilities to control it and, consequently, shift toward a subject position. Thus, the effectiveness of psychotherapy is based on the growth of mentalization capabilities and self-reflection, which then lead to behavioral changes.

Narratively rich game genres, such as role-playing and adventure games, may be particularly suited for adaptations of existing psychotherapies that invite an active reflection on one’s thoughts, emotions, and behaviors. The interactions with game characters can be used to, for instance, simulate challenging social situations through role playing, model effective behaviors, and develop emotional regulation. An example of this approach is SPARX, a serious game for adolescents living with depression. The computerized CBT intervention consists of 7 modules, including psychoeducation, building hope, encouraging behavioral activation, and coping with negative emotions [86].

##### Offering Cognitive Training

Compared with psychotherapeutic approaches, the mechanisms of action of cognitive training games are considerably different. Their rationale lies in the finding that many mental disorders are associated with cognitive deficits [87]. For instance, depression is related to moderate deficits in memory, attention, and executive function, and these deficits may prevail partly even after depression is in remission [88]. Computerized interventions have been designed to address these deficits, and this approach has been found effective in addressing depressive symptoms [89].

Compared with reflective psychotherapeutic games that rely on player interaction with the narrative, characters, and story, the mechanisms of action in cognitive training games are more closely associated with the game mechanics. For instance, fast-paced first-person shooter games, real-time strategy games, and other action games may provide pathways for improving cognitive skills [90] and alleviating cognitive deficits associated with mental health challenges. Furthermore, the mechanisms of action may also be related to game preferences: reflective therapies may be preferred by those with a tendency for immersion and socialization, whereas cognitive training games appeal to achievement, exploration, and domination motives. Bearing in mind that fast-paced games are preferred by younger audiences [31], the interventions may be suited to addressing cognitive deficits associated with mood or neuropsychological disorders in the youth. An example of this approach is EndeavorRx, a gDMHI that seeks to alleviate pediatric ADHD [91].

##### Encouraging Physical Activity

To further contrast the mechanisms of action of reflective psychotherapeutic games and those of cognitive training games, the effectiveness of exergames is dependent on increasing physical activity. The rationale lies in the finding that physical exercise has significant and large antidepressant effects [92,93]. For instance, the augmented reality game Pokémon Go, which encourages finding, gathering, and collecting digital fantasy pets from various real-life locations, has been found to contribute to a greater number of daily steps and increased social interactions because the locations are frequented by like-minded players [94]. These interactions, in turn, may have positive effects on mood [95]. It appears that the interventions that encourage physical activity often use a purpose-shifted approach facilitated by the availability of exergames [2-4,96]. Currently, however, the quality of evidence on the effectiveness of exergames for mental disorders is low.

We highlight how development efforts need to find the synergy between the health research–based mechanisms of action and their game-based implementation. The 3 described mechanisms of action, built on existing psychotherapies, cognitive training, and physical exercise, exemplify how health care research can intertwine with the game genre implementation.

#### Effectiveness: What Does the Intervention Achieve?

The effectiveness of gDMHIs is indicated by behavioral and symptom changes [10,13]. Reaching a meaningful reduction in symptom scores indicates a clear benefit expected by clinicians and the players and payers of the intervention alike. To complement the symptom- or disorder-focused approach, the intervention may have other, equally meaningful benefits that occur in parallel or in addition to the symptom change. As the World Health Organization (WHO) [97] states in its constitution, “health is a state of complete physical, mental and social well-being and not merely the absence of disease or infirmity*.*” In subjective terms, symptom alleviation may be related to improved quality of life [98]. To illuminate the complexity of the concept, a qualitative synthesis of the experiences of people with mental disorders found that good quality of life was associated with feelings of well-being, positive self-perception, control, autonomy, a sense of belonging, participation in meaningful activities, and a positive future view [99]. In other words, an improved quality of life is not perceived merely as the absence of symptoms.

Expanding from the symptom-based approach, the impact of gDMHIs may be considered through the self-determination theory [100]. It describes the following 3 universal human needs that contribute to intrinsic motivation, self-regulation, and well-being: competence, autonomy, and relatedness. Digital games have a unique possibility to contribute to the 3 needs by fostering a sense of competence by presenting the players with increasingly difficult puzzles as their skill increases, creating a sense of connection with other players enjoying similar activities, and supporting player autonomy by offering choices and various ways to play. As opposed to mediums that are more passively consumed, games are characterized by a sense of agency [101]: the player does not spectate the protagonist but becomes them, controls them, and sees the fiction through them. By answering to the players’ needs, game-based interventions can have positive effects across the cognitive, social, and emotional spheres [56].

However, whether the interaction serves the underlying human needs depends on both the game design and the players’ mindset. Games that serve the players’ needs are associated with continued motivation to play [102,103] and affective well-being [55]. When the players use the game to fulfill their psychological needs, playing can be viewed as adaptive and healthy [104], whereas using the game as an avoidance behavior is associated with mental distress and problematic gaming [105]. Thus, creating designs that answer to the players’ deeper psychological needs and encourage healthy playing styles is vital.

User interaction with the gDMHI produces a vast amount of data, including behavioral biomarkers [106], which may contribute toward understanding the psychological needs, mechanisms of action, and mental well-being beyond the disorders. Players’ interaction with the game mechanics and puzzles provides indications of their motor and cognitive performances, which are particularly useful in cognitive training interventions. Players’ affective states and changes in their social interactions may be derived from their text-based outputs and interactions with other players, which contributes to therapies that use reflective approaches. However, the use of behavioral biomarkers as outcome measurements is only growing. Recently, they have been used, for instance, in the assessment of social anxiety [107], mood disorders [108], and mild cognitive impairment [109].

To summarize, gDMHIs are expected to contribute to a significant reduction in mental health symptoms, which is preceded by player engagement and behavior change [13]. Thus, the overall effects of the intervention may extend beyond the symptom change that the digital medium affords to accurately measure. Leveraging in-game data provides corroboratory insights into why and to what extent the interventions are effective, including in terms of broader psychological needs. This contributes to understanding how the interaction impacts a user’s behavior, which is, hopefully, generalized in the broader context of the user’s life [110], exhibiting macrolevel engagement.

### Translating the TEME Framework to Intervention Development

#### Overview

New product development is a gradual, evolving process [10,11]. It can be described in phases, bearing in mind that the research, design, and development efforts, particularly in a novel domain, are often characterized by exploration, iteration, looping back to earlier phases, and redesign based on feedback. Therefore, the development phases should be considered nonlinear.

We summarized gDMHI development into 4 phases (Table 4). The design begins with research into the target audience and their needs, which are performed through a high-level concept that outlines the intervention. The intervention, as well as the service concept more broadly, is advanced iteratively during development. The evaluation of the intervention likewise gradually grows more comprehensive, culminating in a randomized controlled trial (RCT) that is valued as a particularly strong proof in health care. Should the results prove favorable, the solution may be implemented, and its development may continue in live production.

**Table 4:** Throughout the game-based digital mental health intervention development phases, the emphasis shifts among the 4 themes: target audience, engagement, mechanisms of action, and effectiveness. The level of focus on the themes are represented as higher focus (HF) and lower focus (LF).

Theme	Concept design	Development	Evaluation	Production
Target audience: who is the intervention engaging and effective for?	HF	HF	LF	LF
Engagement: do users interact with the intervention?	LF	HF	HF	HF
Mechanisms of action: what explains the intervention effects?	HF	LF	LF	LF
Effectiveness: what does the intervention achieve?	LF	LF	HF	LF

Throughout the 4 phases, the focus of the development team fluctuates between the paradigms of health care and entertainment: designing an effective health care intervention and an engaging video game experience [111], pursuing instrumental and experiential outcomes [39], or, in other words, creating therapeutic value and game value [40]. The 2 complementary perspectives intertwine through the development process.

#### Concept Design: A Model for Addressing the Target Audience’s Needs

The concept provides an overview of *what* the intervention is, which is intimately related to the following question: *who* is it for? Understanding the target audience and their contextual needs and preferences allows defining the characteristics of the intervention [10,11,112]. Interviews, questionnaires, focus groups, desk research, and ethnography can be used to create a rich understanding of the target audience’s preferences, media used, and target audience's thoughts about and frictions with the existing solutions and services. However, the tools are secondary to the aim of shifting one’s perspective through empathy, which is a cornerstone of design [111]. In fact, several development frameworks, models, and philosophies focus on users. They include participatory design [113]; service design [111]; persuasive design [114]; and user-centered design [115], which is also common in gDMHI design. A study exploring 20 development processes found that 50% of them adopted a user-centered approach in the concept phase, and the rest invited user participation in the development phase [116]. Accentuating the importance of users reflects the threats of overly focusing on the solution instead of the need it addresses, which may lead to users rejecting the intervention, and necessitating costly changes later in the development [4,117]. Starting from the target audience, as well as other necessary stakeholders, facilitates the definition of the intervention objectives, technology, and game genre [51].

It is a common misconception that user research would directly inform the design. Users can describe their experiences with existing solutions, but developers are responsible for integrating the understanding into a model that considers the mechanisms of action, technological possibilities, stakeholder dependencies, commercialization, and regulations. Often, the digital concept aims to challenge the dominant, nonscalable, one-to-one, and in-person model of mental health service delivery [118]. The concept can also include considerations of the business model: how the intervention is priced, purchased, and compensated [119]. In contrast to the entertainment industry, which is dominated by the direct-to-consumer model, where the player chooses, uses, and pays for the game, health care characteristically includes more complicated commercial models. Aitken and Nass [32] described 4 such commercial models: direct-to-consumer model, where the user pays for the service; device-like reimbursement model, where the cost of the intervention is covered by the medical benefit plan when the intervention is prescribed by a physician; drug-like reimbursement model, which relates the intervention to pharmacy benefit; and value-based contracting, where the intervention is provided for an organization and paid per the benefits achieved. Thus, the preliminary documented concept is a user-centered account of whom the team is designing for, a science-based account of how the intervention contributes to the user’s health, and a description of the intended commercial model.

#### Development: Iterating the Design and Planning Its Implementation

During development, the team gradually turns the concept into an actual intervention. The phase is iterative: it oscillates between design and testing, which seeks to ensure that the team is progressing in the right direction [10,112,115]. During development, the team can make use of methods from entertainment, which allow the team to focus specifically on the intervention features, and methods from health care, which focus on establishing and implementing the intervention in the complex health care ecosystem.

The success of games is tied to the experience they create [46]. There are various perspectives on game experience [120]; however, in general, it is built of layers, as the user interacts alone or with other players with the game system, its look and feel, and the underlying systems. The interaction leads to an experience that the player may find immersive, motivating, and pleasurable. Entertainment game design has a wealth of methodologies that can be used to hone the game-user interaction [121-123], which acts as a focus point for the interdisciplinary efforts in visual and audio design, software development, and storytelling [44].

The business-to-consumer digital entertainment industry has established distribution platforms that allow developers to focus on game development. Health care, in contrast, currently lacks such global, or even national, distribution platforms. Therefore, it is important to design for feasibility and consider the implementation of the intervention early [11]. If the intervention is introduced into an organizational context, it needs to fit the existing practices, improve current processes, and integrate with the existing software solutions [124]. In fact, many interventions are expected not only to offer end user benefits but also to be easy to adopt and contribute to organizational efficiency. The Non-adoption and Abandonment of technologies, and the challenges to Scale-up, Spread, and Sustainability of such technologies (NASSS) framework structures the complex health care environment in which the intervention may be implemented [125]. It highlights the interrelations among the health condition, technology, value proposition, stakeholders adopting the solution, and their organizational and wider context over time. In conclusion, the development process gradually builds not only the digital intervention, in the narrow sense of the word, but also the related services and the operating models that sustain it.

#### Evaluation: Progressive and Comprehensive Intervention Evaluation

The evaluation of the intervention grows gradually more comprehensive. In development, the intervention is tested for its playability and game experience, ensuring that the game-based intervention is found sufficiently favorable by the players in the target audience [46] and is meaningful for other stakeholders. As the development progresses, there is increasing interest in evaluating the intervention’s health-related effectiveness. A feasibility study, a higher-order concept than playtesting, focuses on the following question: can the intervention work? [126]. Feasibility studies allow for evaluating whether the intervention can attract the appropriate target audience, understanding how acceptable the participants consider the intervention and its procedures, and understanding how the intervention is to manage in practice and contribute to the development of data collection methodologies. However, a feasibility study provides only initial indications of the intervention’s effectiveness. Pilot studies can create stronger, although inconclusive, evidence of intervention effectiveness [126]. Should the intervention pass the preliminary evaluations, an RCT can be conducted. It provides high-quality evidence on the effectiveness and safety of the intervention for regulators, clinical stakeholders, and the scientific community. However, because conducting an RCT is demanding, expensive, and slow, progressing to them without ensuring the overall feasibility of the intervention is not advised [112].

It is important to evaluate the intervention comprehensively beyond the summative results: formative evaluation can provide insights into how and why the intervention is or is not successful in its context [127]. Qualitative research can contribute to understanding the intervention’s impact and player experience [128,129], and evaluating engagement creates insights into the intervention’s feasibility, acceptability, attractiveness, and dose-response relationship. However, the evaluation of engagement is often lacking, and there is substantial heterogeneity in how it is reported [26,73]. One approach is reporting the following 6 factors: the number of users, their profiles, the number of modules in the intervention, the number of times they are accessed, the percentage of users receiving a therapeutic dose, and the achieved clinical change [26]. Economic evaluation is not to be overlooked either, as it often forms the rationale for adopting a digital approach. Economic evaluation can be performed to understand, for instance, the cost-effectiveness of the intervention and to guide strategic decision-making and investments [130].

We encourage a progressive and comprehensive evaluation of the intervention, that is, considering the subjective user experience and objective engagement alongside the clinical metrics. Complementing summative evaluation with formative and economic evaluations allows for an understanding of the intervention feasibility and whether the benefits justify the costs, which facilitates translating the research to complex real-world environments should the intervention prove both effective and engaging.

#### Production: Implementing the Intervention in Real Life

When the intervention works in controlled research settings, it may be implemented in more complex, real-life settings. Following the principle of path dependence, the decisions made in the earlier stages regarding, for instance, the health indication, mechanisms of action, game genre, and technical implementation continue to exercise their influence in the production phase. Building on the established foundation, the attention turns to marketing, service production, partnerships, and continuing development.

Implementing the intervention often encounters challenges: the research-to-practice gap [27]. Common barriers include user preference for face-to-face therapies, the perceived complexity of the digital intervention, limited research evidence regarding the intervention, low user engagement, the costs of the intervention, and practitioners’ resistance. To overcome these challenges and achieve real-world impacts, developers should prepare for them using numerous models [124,125,131]. The Reach, Efficacy, Adoption, Implementation, and Maintenance (RE-AIM) model provides an overview of the components necessary for creating an impact on a population level: the intervention needs to reach a broad audience, be effective, be adopted in numerous settings, be implemented as intended, and be maintained over time [131]. Thus, besides the need for the intervention to be proven effective, the implementation of the intervention demands marketing and organizational support for dissemination. Another useful perspective is the consolidated framework for implementation research, which consists of 5 domains [124]. Intervention implementation is dependent on the intervention characteristics, relationship of the intervention with external stakeholders, organization’s internal capabilities to produce the service, individuals within the organization delivering the intervention, and implementation processes. Outside the research setting, the development team needs to consider ways to deliver the intervention to users en masse, which demands changes in organizational structures and service management.

Should the intervention reach a sufficiently considerable population, the developers may begin to view the software as a continuous service. Here, methodologies already in use in game development may prove useful. In contrast to the earlier, linear development process that considered the launch of the game the conclusion to the development, the prevailing game as a service model focuses attention also on postlaunch development [132]. This approach, also called live operations, relies on using business metrics and user feedback to guide the continuous development efforts and add new content [133]. In the future, there is potential in considering gDMHIs as a continuous, evolving service rather than unaltered products [134]. However, it is still unclear to what degree the digital intervention may be developed while retaining the effects and evidence gathered in the previous stages, and there is little research on gDMHI live operations. This reflects the nascent industry that is still waiting for successful interventions to attract a large number of users to fund the continuous research and development of a live product.

## Discussion

### Summary

gDMHIs use elements of entertainment games to achieve health-related outcomes. We describe how these interventions draw from the health care and entertainment paradigms (Table 1) and categorize them in a continuum between the two (Figure 1). We then introduce 4 themes, the TEME framework (Table 2), to negotiate between the 2 paradigms and facilitate their interdisciplinary development from concept design to production (Table 4).

### Contributions to the Development Research

Previous research has discussed the tension of interventions being both engaging and effective [38-40]. By contrast, the existing development frameworks have facilitated the development of behavioral interventions, digital interventions, and serious games for health [10-13]. We expand on the prior literature by elaborating, contrasting, and negotiating the differences between the paradigms of health care and entertainment. The paradigm of health care frames and measures user needs through diagnoses and behavior, has expansive research on mechanisms of action, and is unrelenting in its focus on proving effectiveness, yet it has a shorter history in crafting and implementing engaging experiences in the digital setting. Meanwhile, digital entertainment leads and pioneers in this field. It has expansive practices in iteratively creating immersive experiences in numerous game genres and has advanced development methodologies to measure and design for subjective and objective engagement. However, the entertainment paradigm lacks an understanding of the health indications, mechanisms of action, and health care ecosystem. Thus, the interdisciplinary gDMHI development requires broad competencies in both paradigms and their methods: the digital game–based medium and the health care context.

The TEME framework supports interdisciplinary collaboration by focusing on 4 themes necessary for development: understanding the target audience, ensuring that the intervention is found favorably by them, basing the intervention on proven mechanisms of action, and ensuring its effectiveness. When the 4 themes are reflected in development, developers can seek synergy between health care research and the game-based medium. Common approaches include adapting existing psychotherapies, offering cognitive training, and encouraging physical exercise. However, there are also other developments to be imagined and researched: perhaps the genre of peaceful and serene walking simulators [135] characterized by the ponderous, slow pace could be used to encourage mindfulness practice, or web-based communities could be used to offer peer support, a sense of belonging, and empowerment [136]. We encourage iterative intervention development and evaluation beyond symptom change, considering the behavioral, economic, and organizational impacts.

Earlier, gamification and serious games were considered 2 distinct lines of research [50]. Taking an integrative stance, we suggest that gamified interventions, serious games, and purpose-shifted digital entertainment games can also be viewed as a continuum between health care and entertainment. This distinction contributes a novel perspective to the classification of serious games and serious games in health [51,53,137], allowing an understanding of the developer’s position between the 2 approaches.

Earlier, Fleming et al [115] called for a paradigm shift in the development of digital interventions. In particular, they suggested increasing efforts in user-centered design, creating engaging interventions, fostering collaboration, and conducting rapid testing and implementation. Building on these principles, we describe how the development of gDMHIs requires using the strengths of both digital entertainment and health care through an integrative, interdisciplinary framework.

### Conclusion: Game-Based Elements Are More Than a Spoonful of Sugar

“In every job that must be done/There is an element of fun/You find the fun and snap!/The job’s a game,” said supernanny Mary Poppins when instructing unwilling children on how to get their chores done. With the mindset shift, the mislaid items find their place through the magic of motivation. Similarly, the effectiveness of a gDMHI depends on the design’s ability to conjure motivation and identification with the change in the users. Engagement and effectiveness depend on the target audience, the underlying mechanisms of action, and their execution as a game. When successful, the game-based approach is more than adding a spoonful of sugar to sweeten an otherwise uninteresting task. Rather, it allows creating interventions that bridge the treatment, engagement, and implementation gaps: they allow expanding intervention reach, inspire effective engagement, and allow viable intervention production.

## Abbreviations

ADHD: attention-deficit/hyperactivity disorder
COM-B: capability, opportunity, motivation, and behavior
DMHI: digital mental health intervention
gDMHI: game-based digital mental health intervention
NASSS: Non-adoption and Abandonment of technologies, and the challenges to Scale-up, Spread, and Sustainability of such technologies
RCT: randomized controlled trial
RE-AIM: Reach, Efficacy, Adoption, Implementation, and Maintenance
TEME: target audience, engagement, mechanisms of action, and effectiveness
WHO: World Health Organization
